# Supplementary material for: Increased Dietary Trp, Thr, and Met Supplementation Improves Performance, Health, and Protein Metabolism of Weaned Piglets under Mixed Management and Poor Housing Conditions
Source: Animals (Basel). 2024 Apr 9;14(8):1143. doi: 10.3390/ani14081143 (PMC11047353; doi:10.3390/ani14081143)
Supplement: Supplementary file 1 [file animals-14-01143-s001.zip › SM.pdf]

Supplementary Material

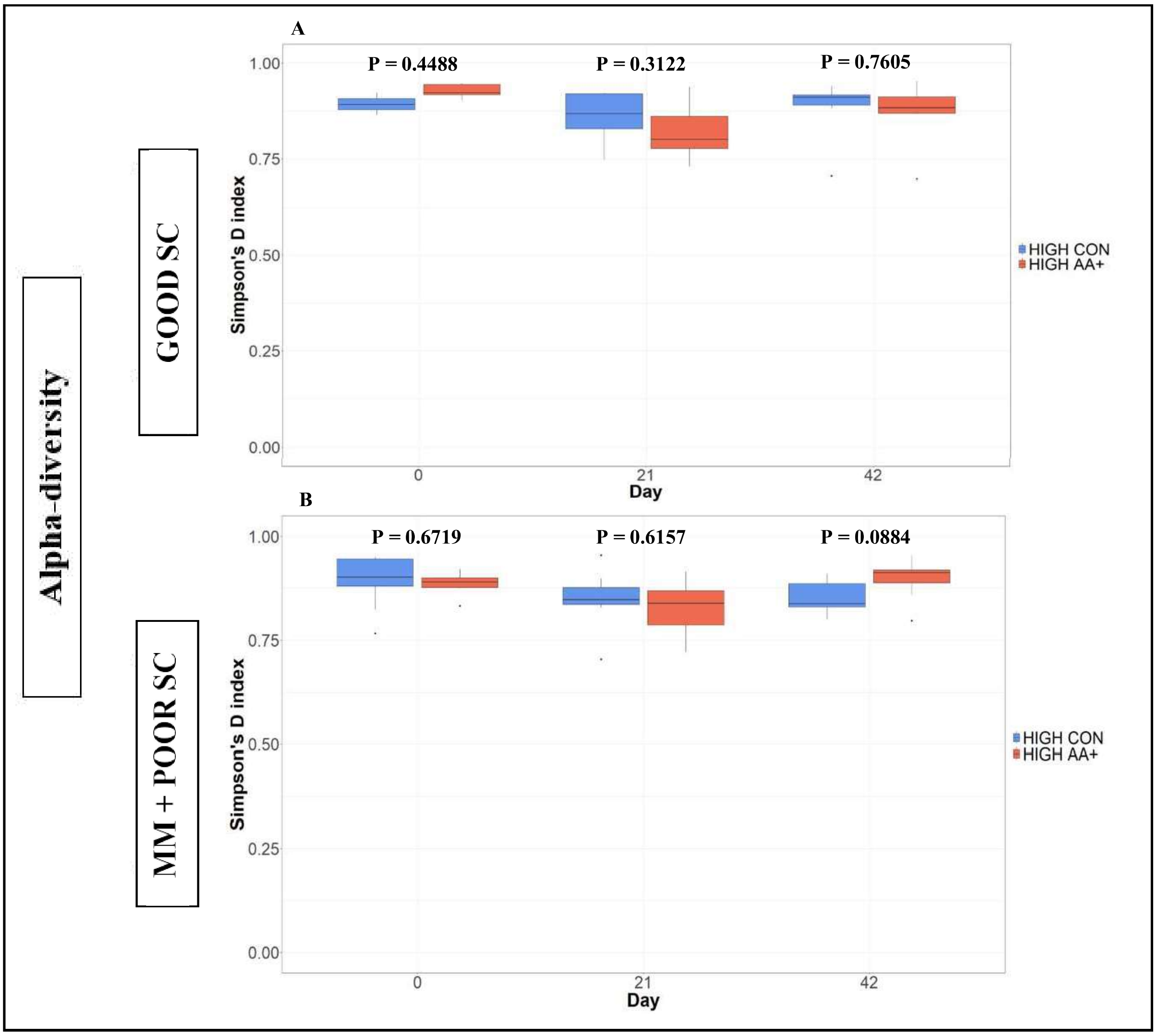

Figure S1. Alpha-diversity (Simpson's D index) analysis of fecal microbiome samples across piglets with High Sanitary Status (HSS), housed in a good or poor sanitary condition (SC), and feeding a control (CON) or surplus supplemented diet with amino acids (AA+). Figure S1A shows no significant difference in alpha-diversity (Simpson's D Index) between diets (CON vs. AA+) across HSS piglets under GOOD SC over time. Figure S1B shows no significant difference in alpha-diversity (Simpson's D Index) between diets (CON vs. AA+) across HSS piglets under mixed management and poor sanitary condition (MM+POOR SC) over time. Alpha-diversity comparison between diet groups was done using a Welch two sample T-test ($P < 0.05$). The sample size for the fecal samples was: **GOOD SC** (Figures S1A) on day 0, CON (n=2) and AA+ (n=5); on day 21, CON (n=7) and AA+ (n=7); and on day 42, CON (n=8) and AA+ (n=8); and **MM + POOR SC** (Figures S1B) on day 0, HSS CON (n=10), HSS AA+ (n=7); on day 21, HSS CON (n=7), HSS AA+ (n=7); and on day 42, HSS CON (n=7), HSS AA+ (n=8). Each piglet was considered SC an experimental unit throughout the analysis.

Table S2 - Serological results of the health monitoring program of the high and low sanitary status farms.

Characteristics	High Sanitary Status	Low Sanitary Status
Seronegative to antibodies against:	*Mycoplasma hyopneumoniae*, *Actinobacillus pleuropneumoniae* (APP), Swine InfluenzaH1N1	
Seropositive to antibodies against:	Porcine circovirus type 2, *Glaesserella parasuis*, *Pasteurella multocida*, *Lawsoniaintracellularis*	*M. hyopneumoniae*, *Actinobacillus pleuropneumoniae* (APP), Swine Influenza H1N1, Porcine circovirus type 2, *Glaesserella parasuis*, *Pasteurella multocida*, *Lawsonia intracellularis*
