# Supplementary material for: Increased Dietary Trp, Thr, and Met Supplementation Improves Performance, Health, and Protein Metabolism of Weaned Piglets under Mixed Management and Poor Housing Conditions
Source: Animals (Basel). 2024 Apr 9;14(8):1143. doi: 10.3390/ani14081143 (PMC11047353; doi:10.3390/ani14081143)
Supplement: Supplementary file 1 [file animals-14-01143-s001.zip › Table S1_Animals.pdf]

Table S1. Forward and reverse sequence reads per samples.

sample ID	forward sequence count	reverse sequence count
D0JA5-3A2	68940	68940
D0JA203	60591	60591
D0JA227	60468	60468
D0JA46	57553	57553
D0JA220	53467	53467
D0JA25	53195	53195
D0JA116	52356	52356
D0JA5	52071	52071
D0JA52	50577	50577
D0JA250	50091	50091
D0JA81	49157	49157
D0JA219	44563	44563
D0JA107-3A9	44315	44315
D0JA215	42769	42769
D0JA141	42514	42514
D0JA107	42229	42229
D0JA101-3A8	36135	36135
D0JA230	32176	32176
D0JA101	32093	32093
D0JA231	32086	32086
D0JA130	32012	32012
D0JA10	30220	30220
D0JA55	30038	30038
D0JA114	29678	29678
D0JA206	29259	29259
D0JA62	27582	27582
D0JA79	27323	27323
D0JA114-3A10	27128	27128
D0JA116-3A11	26647	26647
D0JA133	26318	26318
D0JA205	25863	25863
D0JA105	25314	25314
D0JA51	22257	22257
D0JA217	22075	22075
D0JA128	19478	19478
D0JA210	18062	18062
D0JA12	17214	17214
D0JA127	16696	16696
D0JA2	15448	15448
D0JA73	14583	14583
D0JA57	3155	3155
D0JA140	15148	15148
D21JA219	39689	39689
D21JA51	37390	37390
D21JA101	36060	36060
D21JA107	34489	34489
D21JA116	33992	33992
D21JA250	32162	32162
D21JA2	31374	31374
D21JA203	30058	30058
D21GA92	29289	29289

D21JA127	26281	26281
D21JA15	25362	25362
D21JA30	25069	25069
D21JA230	24642	24642
D21JA114	23359	23359
D21JA231	23201	23201
D21JA5	22889	22889
D21JA9	22759	22759
D21GA29	22249	22249
D21JA217	22063	22063
D21JA10	22053	22053
D21JA139	19997	19997
D21JA45	19777	19777
D21JA215	17528	17528
D21JA81	16666	16666
D21JA57	16053	16053
D21JA25	15767	15767
D21JA206	15537	15537
D21JA55	15497	15497
D21JA140	14852	14852
D21JA13	14450	14450
D21JA105	13437	13437
D21JA205	12114	12114
D21JA90	11561	11561
D21JA69	11244	11244
D21JA62	10647	10647
D21JA52	9998	9998
D21JA227	9432	9432
D21JA141	8765	8765
D21JA220	7566	7566
D21JA128	6844	6844
D21JA46	6367	6367
D21JA84	5252	5252
D21JA210	4601	4601
D21JA112	36308	36308
D21JA133	33593	33593
D21JA141-B9	10703	10703
D42JA60	54075	54075
D42JA5	49864	49864
D42JA20	43912	43912
D42JA81	41242	41242
D42JA2	32775	32775
D42JA62	32182	32182
D42JA203	30863	30863
D42JA69	30155	30155
D42JA227	29397	29397
D42JA15	29169	29169
D42JA133	28779	28779
D42JA107	28245	28245
D42JA46	27214	27214
D42JA219	26774	26774
D42JA141	25270	25270
D42JA215	25075	25075

D42JA9	23988	23988
D42JA205	23827	23827
D42JA112	22523	22523
D42JA6	21543	21543
D42JA210	21352	21352
D42JA55	21121	21121
D42JA249	21033	21033
D42JA45	20719	20719
D42JA250	20110	20110
D42JA13	20045	20045
D42JA101	19398	19398
D42JA140	19109	19109
D42JA128	18702	18702
D42JA124	17643	17643
D42JA116	15423	15423
D42JA139	15328	15328
D42JA220	14855	14855
D42JA57	14793	14793
D42JA206	14370	14370
D42JA105	14121	14121
D42JA90	12564	12564
D42JA84	12356	12356
D42JA95	9166	9166
D42JA217	9024	9024
D42JA22	8959	8959
D42JA127	6839	6839
D42JA231	5630	5630
D42JA52	16942	16942
sum	3384304	
average	25638,66667	
